# Utilization of vinasse for production of poly-3-(hydroxybutyrate-co-hydroxyvalerate) by *Haloferax mediterranei*

**DOI:** 10.1186/2191-0855-2-34

**Published:** 2012-07-09

**Authors:** Anirban Bhattacharyya, Arnab Pramanik, Sudipta Kumar Maji, Saubhik Haldar, Ujjal Kumar Mukhopadhyay, Joydeep Mukherjee

**Affiliations:** 1 School of Environmental Studies, Jadavpur University, Kolkata, 700 032, India; 2 Department of Chemistry, Jadavpur University, Kolkata, 700 032, India; 3 West Bengal Pollution Control Board, Paribesh Bhavan, 10A, Block LA, Sector III, Salt Lake City, Kolkata, 700 098, India

**Keywords:** Poly-3-(hydroxybutyrate-co-hydroxyvalerate), Vinasse, Polyphenols, Haloarchaea, *Haloferax mediterranei*

## Abstract

Vinasse, a highly polluting waste of the ethanol industry was utilized for the production of polyhydroxyalkanoate (PHA) by the extremely halophilic archaeon, *Haloferax mediterranei* in shake-flasks. Following pre-treatment through adsorption on activated carbon, 25%-50% (v/v) pre-treated vinasse was utilized leading to 70% maximum accumulation of PHA. Maximum PHA concentration of 19.7 g/l, product yield coefficient (based on total carbohydrates) of 0.87 and 0.21 g/l h volumetric productivity were achieved. Concomitant lowering of BOD_5_ of pre-treated vinasse by at least 78% and COD by at least 80% was attained at the end of this process. The PHA was recovered by osmotic lysis of the cells and purification by sodium hypochlorite and organic solvents. Through UV–vis spectroscopy, gas chromatography, differential scanning calorimetry and nuclear magnetic resonance spectroscopy, the PHA was identified as poly-3-(hydroxybutyrate-co-hydroxyvalerate). The 3-hydroxyvalerate content was 12.36 mol % (utilizing 25% pre-treated vinasse) and 14.09 mol % (utilizing 50% pre-treated vinasse). High salt concentration in the medium allowed this process without sterile conditions and thus reduction in costs of sterilization can be envisaged. Activated charcoal pre-treatment of vinasse is economical than competing processes such as ultrafiltration of whey, extrusion and enzymatic treatment of rice and corn starch. Without impacting sugar prices, this process can easily be integrated into a distillery that has fermentation equipment and trained personnel. High PHA content, productivity, zero-cost carbon source, low-cost isolation of a high-purity product and potential integration into ethanol manufacturing unit with concomitant wastewater treatment should merit further development of this process to higher scales.

## Introduction

Polyhydroxyalkanoates (PHAs) are natural, renewable and biocompatible biopolymers which can be made into plastic materials with properties similar to petrochemical plastics. Produced intracellularly in bacteria, PHAs can replace synthetic plastics in many applications. However, high production cost of the biopolymers and concurrent availability of low-cost petrochemical-derived plastics make PHA manufacture economically unattractive. To make the PHA production financially more appealing, the use of inexpensive substrates has been the subject of many investigations (Verlinden et al. [2011]).

The effluents from molasses based distilleries contain large amounts of dark brown colored molasses spent wash or vinasse. In the distillation process, ethanol ranges from 5% to 12% by volume, hence it follows that the amount of waste varies from 88% to 95% by volume of the alcohol distilled. An average molasses based distillery generates 15 l of vinasse per liter of alcohol produced. Vinasse is one of the most difficult waste products to dispose because of low pH, high temperature, dark brown color, high ash content and high percentage of dissolved organic and inorganic matter (Pant and Adholeya, [2007]). In attempts to utilize vinasse as a low cost carbon source for commercial synthesis of PHAs, polyhydroxybutyrate (PHB) was produced from spent wash obtained from a distillery producing ethanol by fermentation of sugarcane molasses (Khardenavis et al. [2009]) as well as rice grain and jowar grain based distillery vinasse (Khardenavis et al. [2007]) by mixed culture of microbes.

Halophilic microorganisms are an important source of PHAs and hold promise for providing an economically competitive industrial scale production process. Haloarchaeal strains employed as PHA producers have many advantages. As there are very few microorganisms that are able to survive and grow at high salinities, the risks of microbial contamination can be reduced. Haloarchaea can be easily lysed in distilled water, therefore, the use of large quantities of organic solvents can be avoided and time for PHA preparation can be saved. Cheap carbon sources can be used to synthesize PHAs by haloarchaea and so the cost of PHA production will be lowered (Quillaguamán et al. [2010]).

In our previous article (Pramanik et al. [2012]), we provided the first report of PHB production by a halophilic microorganism, *Haloarcula marismortui* utilizing vinasse. Our long term objective is to expand the possibility of PHA production by other extreme halophiles consuming vinasse. Thus the specific questions to be answered in this study were: can the methods developed be applied for the production of biopolymers other than PHB? Is the product obtained of high purity? Is reasonable yield and productivity achieved? Although the family Halobacteriaceae includes 30 genera, currently, only a few haloarchaeal strains belonging to the genera *Haloferax*, *Haloarcula*, *Haloquadratum*, *Haloterrigena*, *Halorhabdus*, *Halobiforma* and *Halopiger* are found to accumulate PHAs. *Haloferax mediterranei* is so far the best PHA producer of the family Halobacteriaceae. The genes, molecular basis and functional genomics of PHA synthesis by *H. mediterranei* and related archaea were studied by Xiang’s research group (Liu et al. [2011] and relevant references therein). Therefore, it was our objective to cultivate *H. mediterranei* in vinasse, isolate the polymer and study its properties.

Due to its high growth rate, metabolic versatility and genetic stability *H. mediterranei* has become an interesting microorganism for investigating PHA production. The PHA accumulated by *H. mediterranei* (PHBV), has much better mechanical properties than PHB, (the most frequently occurring PHA) and hence is more promising for commercial production and application. Furthermore, *H. mediterranei* can accrue PHBV up to 60% (wt/wt) from starch, glucose or other cheaper industrial by-products without the addition of costly and cellular toxic carbons, such as propionic acid or valeric acid that are generally required by producing eubacteria as precursors of the 3-hydroxyvalerate (HV) unit. Therefore, *H. mediterranei* has become one of the most potential candidate organisms for industrial PHA production (Lu et al. [2008]).

## Materials and methods

### Pre-treatment of vinasse

The vinasse used in this study was obtained from IFB Agro Industries, Noorpur, India that produces ethanol from sugarcane molasses. One hundred milliliters raw vinasse was adjusted to pH 2.0 and treated with 5.0 g activated carbon (AC) as described in Pramanik et al. ([2012]). The AC was removed by centrifugation, the supernatant filtered, neutralized with NaOH/HCl as required and the filtrate characterized as mentioned in the sub-section, “Analytical Methods”.

### Microorganism

*Haloferax mediterranei* DSM 1411 (purchased from DSMZ, Germany) was used in this study.

### Evaluation of inhibitory effect of vinasse

Raw vinasse at 10%, 25%, 50%, 75% and 100% concentrations (% content in water, v/v) and pre-treated vinasse at 25%, 50%, 75% and 100% concentrations were used to study the inhibitory effect. Petri plates were prepared containing the growth medium (GM) as suggested by DSMZ, composed of (g/l) NaCl 200.0; MgSO_4_.7H_2_O 20.0; KCl 2.0; casamino acids 5.0; yeast extract 5.0; C_5_H_8_NNaO_4_ 1.0; Na_3_C_6_H_5_O_7_ 3.0; FeCl_2_.4H_2_O 0.036; MnCl_2_.4H_2_O 0.00036; pH 7.0-7.2, swabbed with *H. mediterranei* culture containing 10^8^ CFU/ml and further evaluated as described in Pramanik et al. ([2012]).

### Production of PHA

*H. mediterranei* was cultivated in 100 ml of GM in a 250 ml Erlenmeyer flask for 4 days at 37 °C with shaking at 180 rpm. To develop a concentrated inoculum, the culture was centrifuged at 10000 rpm for 12 min. Inoculum (1.0 ± 0.05 g wet cell mass) was transferred into 100 ml of 25% and 50% pre-treated vinasse to which the other components of MST medium (Lu et al. [2008]) except glucose were added (g/l) NaCl 200.0; MgSO_4_.7H_2_O 20.0; KCl 2.0; C_5_H_8_NNaO_4_ 1.0; KH_2_PO_4_ 0.0375; FeSO_4_.7H_2_O 0.05; yeast extract 1.0. The medium pH was adjusted to 7.2 and was not sterilized. Production was carried out in 250 ml Erlenmeyer flasks by shaking at 180 rpm for 5 days at 37°C.

### Determination of cell dry weight (CDW)

One milliliter of the liquid culture obtained at the end of the process was centrifuged at 10000 rpm and further tested as described in Pramanik et al. ([2012]).

### Isolation of PHA

Broth containing *H. mediterranei* cells was centrifuged at 10000 rpm (Eppendorf model 5810R, rotor F-34-6-38) for 12 min. The pellet was suspended in distilled water with 0.1% sodium dodecyl sulfate (SDS) for 24 hours (Escalona et al. [1996]). The lyzed suspension was centrifuged at 10000 rpm for 12 min and the process repeated twice. A white colored substance was obtained that was dried till constant weight. The white dust was dissolved in 30% sodium hypochlorite solution and the mixture incubated at 30°C for 3 min ([Dong and Sun 2000]). The mixture was centrifuged at 10000 rpm for 12 min, washed with distilled water and 1:1 solution of acetone and ethanol. The pellet was dissolved in 10 ml of boiling chloroform, which was subsequently evaporated and recycled. The undissolved material was removed by filtration (Fernandez-Castillo et al. [1986] and Tamboli et al. [2010]).

### Determination of PHA purity, content and recovery

The purity of the PHA was measured by the crotonate assay ([Law and Slepecky 1961]) and gas chromatography (GC) analysis (Divyashree et al. [2009]). For the crotonate assay, the residue obtained as described before was hydrolyzed and dehydrogenated with concentrated sulfuric acid to obtain crotonic acid, which was quantified by its A_235_. The purity of the sample was calculated from the standard curve obtained using standard P(HB-co-HV) purchased from Sigma-Aldrich, (USA) containing 12 mol% of HV. The UV–vis spectra of the crotonic acid obtained from standard PHBV and our isolated product were acquired with a PerkinElmer Lambda 25 spectrophotometer and compared. For GC analysis, PHA sample (5 mg) was subjected to methanolysis in sealed tubes at 100°C for 140 min. For methanolysis, 1 ml of chloroform, 0.85 ml of methanol, and 0.15 ml of concentrated H_2_SO_4_ were used. Before analysis, 0.5 ml distilled water was added to the sample and contents were shaken vigorously for 1 min. The methyl esters in the organic phase were analyzed by GC with flame ionization detector, in a 25 m PE-5 (fused silica gel–polymethyl siloxane) capillary column (internal diameter 0.20 mm and film thickness 0.33 μm). Organic phase (2 μl) was analyzed with split of 10 ml/min. Helium (0.7 ml/min) was used as a carrier gas. The injector and the detector were at 230°C and 220°C respectively. The program used was: 80°C for 4 min; ramp of 8°C per min up to 10 min; 160°C for 6 min. Calibration was performed using standard PHBV (Sigma-Aldrich, USA) containing 12 mol% of hydroxyvalerate with benzoic acid as internal standard. PHA content was determined by subjecting 10 mg of lyophilized cells obtained at the fourth day (when maximum growth was reached) to methanolysis and GC analysis as described. From a known amount of PHA in the biomass, the percent PHA recovered was calculated based on the purity of the total mass of the sample recovered at the end of the separation process.

### Characterization of PHA

The content of HV comonomer units was determined by GC and standard PHBV with 12 mol % HV was used for comparison. Mol % of 3-HV = (% polyhydroxyvalerate (PHV) in sample PHBV)/(% PHB in sample PHBV + % PHV in sample PHBV), where, % PHB in sample PHBV = SaA_1_/StA_1_ × SaA_3_/StA_3_ × W_1_/W_2_ × 0.88 and % PHV in sample PHBV = SaA_2_/StA_2_ × SaA_3_/StA_3_ × W_1_/W_2_ × 0.12 where StA_1_ = peak area of standard PHB in GC chromatogram, StA_2_ = peak area of standard PHV, StA_3_ = peak area of standard benzoic acid, SaA_1_ = peak area of sample PHB, SaA_2_ = peak area of sample PHV, SaA_3_ = peak area of sample benzoic acid, W_1_ = amount of sample (mg) and W_2_ = amount of standard (mg). Differential scanning calorimetry (DSC) and ^1^ H NMR spectroscopy of the PHA were done as described in Pramanik et al. ([2012]).

### Analytical methods

The pH, BOD_5_, COD, phosphate, total Kjeldahl nitrogen content, total polyphenolic compounds, total carbohydrate, total organic carbon and conductivity of vinasse were measured as described in Pramanik et al. ([2012]). The spent medium after PHA production was also analyzed by measuring the parameters.

## Results

### Pre-treatment and characterization of vinasse

Reductions in total polyphenolic content as well as the other parameters were obtained following pre-treatment at pH 2.0 with 5.0 g activated carbon per 100 ml raw vinasse. We observed concomitant reduction of organic carbon content and BOD_5_ value with removal of polyphenolic compounds (Table 1). Thus, the pre-treatment process lowered the pollution potential of the vinasse. Although the carbohydrate content decreased considerably, the amount was still high enough to support growth and PHA production by *H. mediterranei.*

**Table 1:** Characteristics of raw, pre-treated (at pH 2.0 with 5.0 g activated carbon /100 ml raw vinasse) vinasse and spent medium after bioprocessing

***Parameter***	***Raw vinasse***	***After pre-treatment***		***After bioprocessing***	
		***25% pre-treated***	***50% pre-treated***	***25% pre-treated***	***50% pre-treated***
pH	3.5 ± 0.5	4.0 ± 0.5	4.0 ± 0.5	8.2 ± 0.5	8.1 ± 0.5
BOD_5_ (mg/l)	78170 ± 120	8467 ± 35	15275 ± 38	1800 ± 27	2250 ± 45
COD (mg/l)	90837 ± 45	13950 ± 84	23134 ± 62	2690 ± 17	3850 ± 27
Salinity (mS/cm)	28.5 ± 0.4	7.2 ± 0.2	13.5 ± 0.5	415 ± 11	445 ± 20
Total organic carbon (mg/l)	16540 ± 35	2764 ± 35	6860 ± 54	305 ± 29	359 ± 43
Total carbohydrate (mg/l)	112581 ± 125	22637 ± 87	32935 ± 71	409.5 ± 25.5	604.5 ± 55
Total Kjeldahl nitrogen (mg/l)	471 ± 15	97 ± 18	175.5 ± 22	14 ± 1.5	17 ± 2
Phosphate (mg/l)	2875 ± 10	468 ± 31	938 ± 91	154 ± 12	195 ± 9
Total polyphenolic compounds (mg/l)	667 ± 10	21 ± 0.5	48 ± 1.5	16 ± 2	39 ± 5

The mean values of three determinations in duplicate sets are reported.

### Inhibitory effect of vinasse on *H. mediterranei*

**Figure 1:**
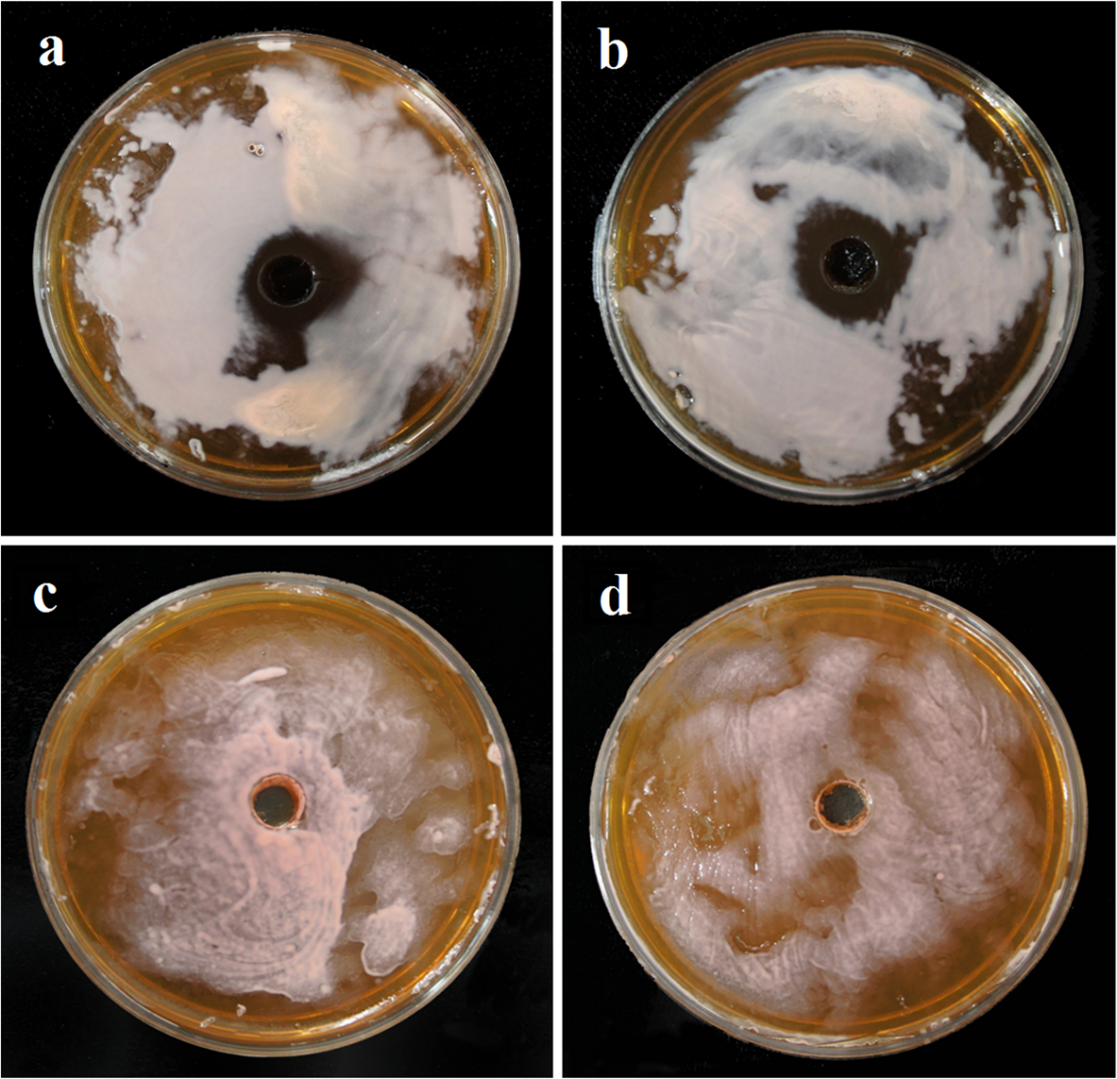
** Antagonistic activity of raw vinasse at concentrations, (a) 25%, (b) 50% and antagonistic activity of pre-treated vinasse at concentrations (c) 25%, (d) 50% against*Haloferax mediterranei*.** Determinations were made thrice in duplicate sets.

10% raw vinasse concentration had no antagonistic effect on the growth of the producer archaebacterium. Higher concentrations of the raw vinasse (25%, 50%, 75% and 100%) inhibited the growth of *H. mediterranei* in increasing extents. However, the pre-treated vinasse allowed the growth of the producer microorganism at 25% and 50% concentrations that inhibited growth in the raw (untreated) form (Figure 1). Growth inhibition of *H. mediterranei* was observed with 75% and 100% pre-treated vinasse and were therefore not selected for further studies.

### Production of PHA using pre-treated vinasse

Referring to Table 1, there was significant lowering of all the measured wastewater parameters of vinasse at the end of the process carried out with 25% and 50% pre-treated vinasse containing medium. Substantial amounts of carbohydrates in vinasse provided the necessary carbon source for the production of PHA. Table 2 shows the bioprocess kinetic parameters determined at the end of the process for growth and PHA production by *H. mediterranei* using pre-treated vinasse. The PHA content and maximum specific growth rates were comparable to those obtained using whey as the substrate. Compared to the processes reported earlier, higher values of PHA concentration, product yield coefficient and volumetric productivity were obtained. Better process performance could be attributed to efficient utilization of the substrate by a concentrated inoculum that led to greater biomass formation. The specific production rate was slightly lower than that of Koller et al. ([2007]) possibly due to the longer process duration. 

**Table 2:** **Kinetic parameters during growth and PHA production by*H. mediterranei*using pre-treated (at pH 2.0 with 5.0 g activated carbon /100 ml raw vinasse) vinasse compared with those obtained previously by cultivating*H. mediterranei*in whey lactose**

***Cultivation***	***PHA (g/l)***	***PHA/CDW (%)***	***μ***_***max***_***(1/h)***	***q***_***p***_***(mg/g h)***	***Y***_***P/S***_	***Volumetric productivity (g/l h)***
25% pre-treated vinasse	19.7	70	0.13	7.3	0.87	0.21
50% pre-treated vinasse	17.4	66	0.12	6.9	0.52	0.18
Whey lactose (Koller et al. [2008])	12.2	73	0.10	150*	0.29**	0.09
Whey lactose (Koller et al. [2007])	5.5	50	0.11	9.1	0.33**	0.05

Time of growth using pre-treated vinasse was 96 h. Notations - *μ*_*max*_: maximum specific growth rate of *H. mediterranei*, *q*_p_: specific production rate of PHA, *Y*_*P/S*_: yield coefficient of PHA based on total carbohydrate in 25% pre-treated vinasse (22637 mg/L) and in 50% pre-treated vinasse (32935 mg/L). * Maximum value; ** in terms of whey sugars.

### Isolation and characterization of PHA

**Figure 2:**
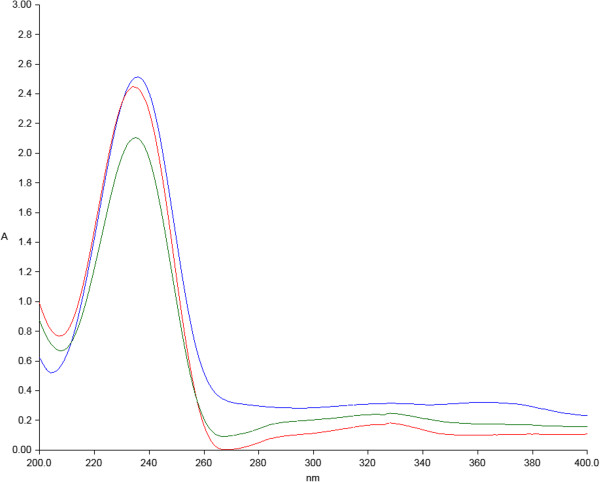
** UV–vis scanning spectroscopy following hydrolysis and dehydrogenation of standard poly-3-(hydroxybutyrate-co-hydroxyvalerate) purchased from Sigma-Aldrich, (USA) containing 12 mol% of hydroxyvalerate (red line) and PHA obtained from*Haloferax mediterranei*cultivated in 25% pre-treated vinasse (blue line) and in 50% pre-treated vinasse (green line).**

**Figure 3:**
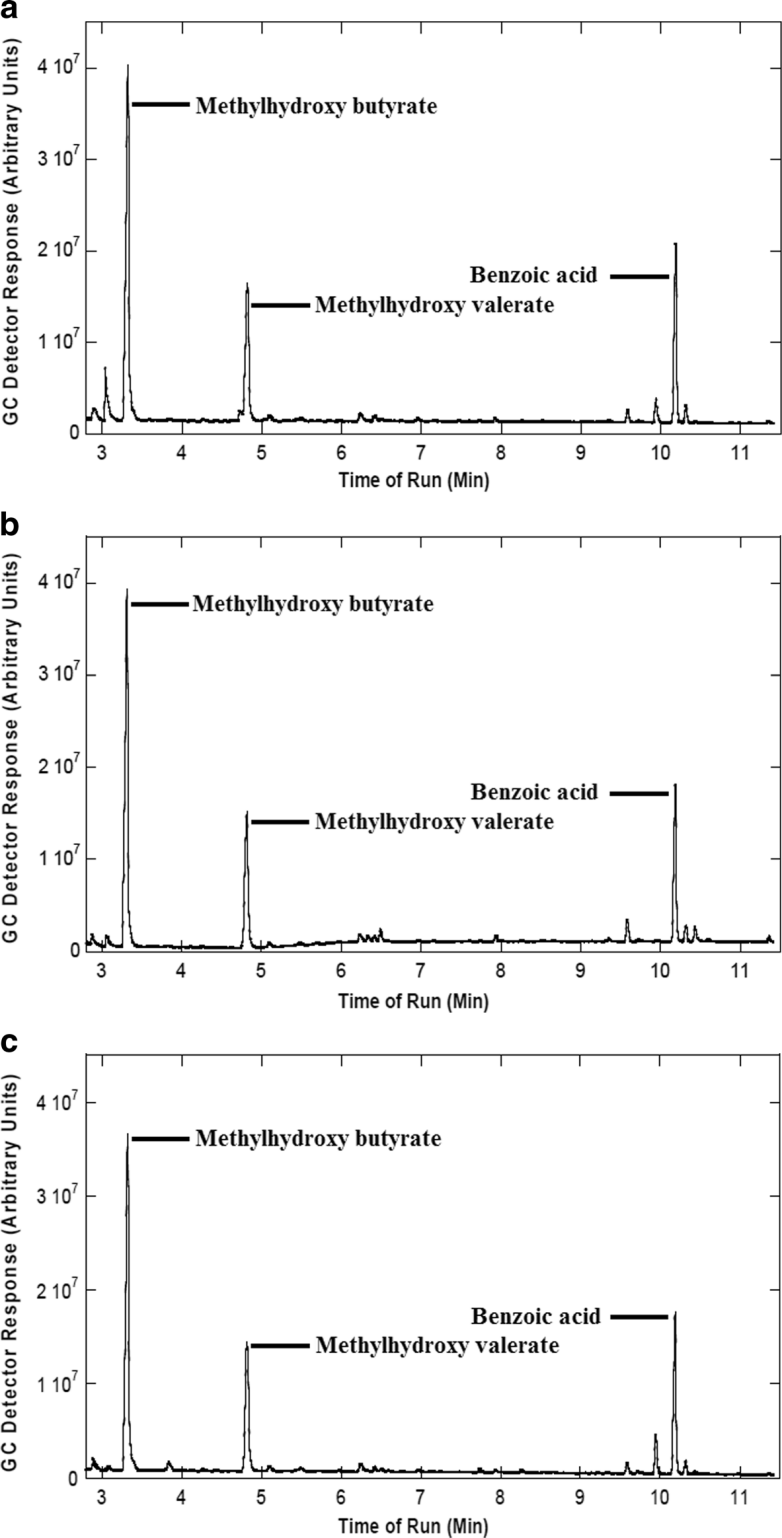
** Gas chromatogram of (a) standard poly-3-(hydroxybutyrate-co-hydroxyvalerate) purchased from Sigma-Aldrich, (USA) containing 12 mol% of hydroxyvalerate (b) PHA obtained from*Haloferax mediterranei*cultivated in 25% pre-treated vinasse and (c) PHA obtained from*Haloferax mediterranei*cultivated in 50% pre-treated vinasse.**

**Figure 4:**
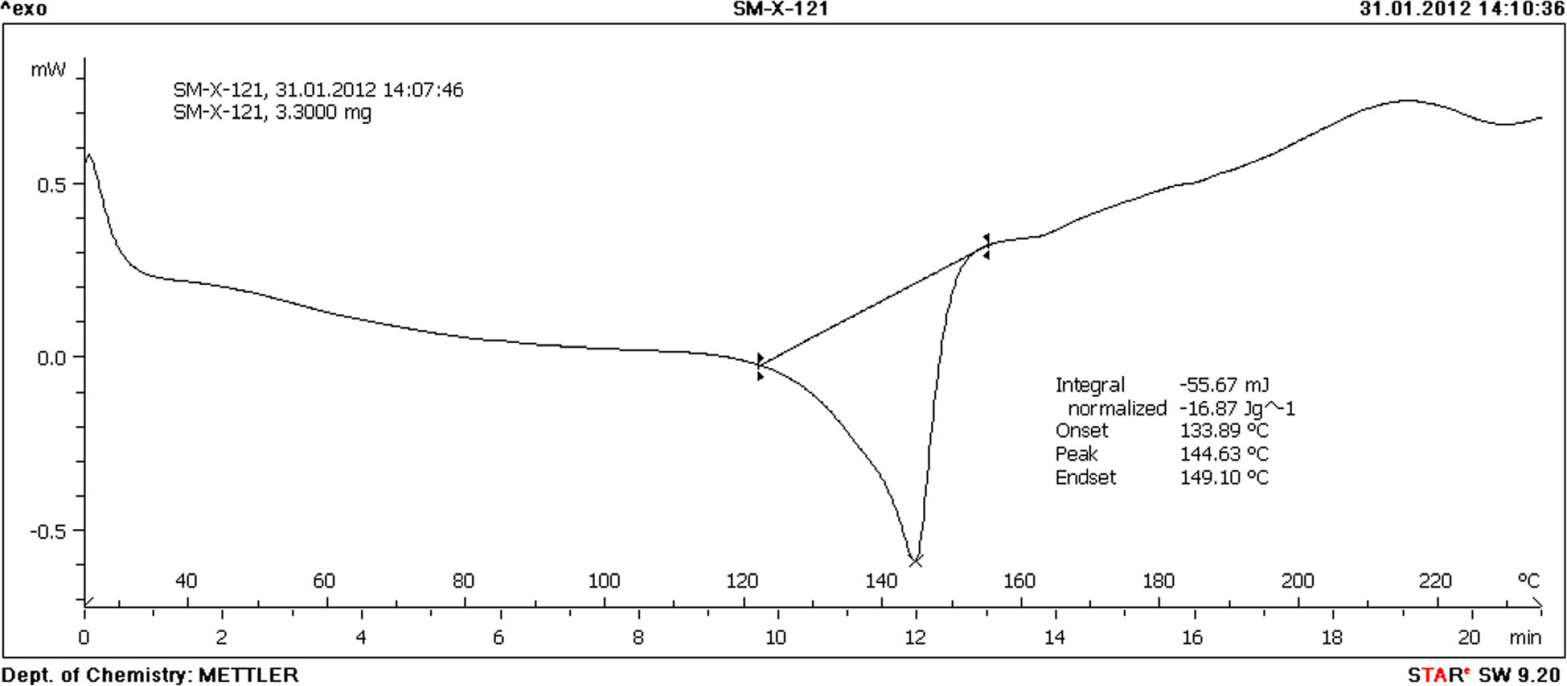
** Differential scanning calorimetry thermogram of PHA obtained from*Haloferax mediterranei*cultivated in 25% pre-treated vinasse.**

**Figure 5:**
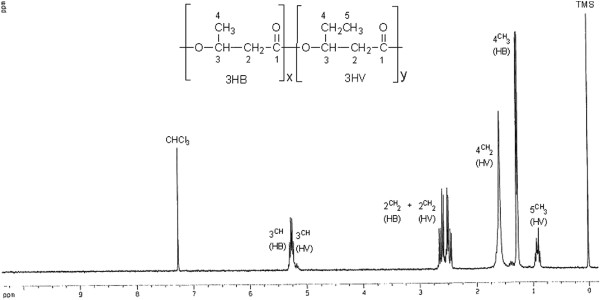
^** 1**^**H NMR spectrum of PHA obtained from*Haloferax mediterranei*cultivated in 25% pre-treated vinasse.**

The recovery of the white mass obtained from *H. mediterranei* cells grown in medium containing 25% and 50% pre-treated vinasse was 97% and 96% respectively. The PHA obtained were ~ 100% pure alike the Sigma-Aldrich product as evidenced by the crotonate assay (Figure 2) and GC analysis (Figure 3). The PHA obtained using 25% pre-treated vinasse contained 12.36 mol % 3-HV while the hydroxyvalerate content was slightly higher (14.09 mol %) in the PHA produced utilizing pre-treated 50% vinasse (Table 3). As apparent from the DSC thermogram (Figure 4), the melting peak was observed at 144.63 °C similar to that recorded at 144.66 °C by Don et al. ([2006]) for PHBV isolated from *H. mediterranei* ATCC 33500 (=DSM 1411). ^1^ H-NMR (CDCl_3_, 300 MHz): δ (ppm): 0.86-0.95 (m, –CH_3_, HV side group), 1.26-1.28 (m, –CH_3_, HB side group), 1.586 (m, –CH_2_, HV side group), 2.430-2.645 (m, –CH_2,_ HV and HB bulk structure), 5.22-5.28 (m, –CH , HV and HB bulk structure). The ^1^ H-NMR spectral data of the PHA obtained by cultivating *H. mediterranei* in vinasse matched with the ^1^ H-NMR spectrum of PHBV acquired by Bloembergen et al. ([1989]) and Liu et al. ([2010]). The multiplet (m) proton signal at 0.86-0.95 region and doublet (d) proton signal at 1.26-1.28 region are the characteristic peaks of methyl (CH_3_) from the hydroxyvalerate unit and methyl (CH_3_) from hydroxybutyrate unit, respectively. The multiplet proton signal at 1.586 ppm is assigned for the γ-CH_2_ proton of HV unit of the polymer. The α-CH_2_ protons for both HB and HV unit appear as a multiplet near 2.430-2.645 ppm, whereas the β-CH protons from either of the units yield a multiplet near 5.22-5.28 ppm (Figure 5). 

**Table 3:** **Contents of 3-hydroxyvalerate in poly-3-(hydroxybutyrate-co-hydroxyvalerate) produced by*Haloferax mediterranei*utilizing vinasse**

***Test compound***	***Amount (mg)***	***PHB peak area (Arbitrary units)***	***PHV peak area (Arbitrary units)***	***Benzoic acid peak area (Arbitrary units)***	***mol % HV***
Standard PHBV (Sigma-Aldrich Cat. No. 403121-10 G)	5.0	983639.33	404768.50	388177.10	12.00
Sample produced from 25% pre-treated vinasse	4.8	926872.73	394958.50	323902.10	12.36
Sample produced from 50% pre-treated vinasse	4.7	879396.30	372125.40	325204.50	14.09

## Discussion

Vinasse is extremely polluting due its high organic load and antibacterial activity which arises from the presence of phenolic compounds (Martín Santos et al. [2003]) that inhibit the PHA producing microorganism, *H. mediterranei.* Thus, pre-treatment for removal of polyphenolic compounds was necessary and the process proved to be effective as 25-50% of an acutely polluting waste like vinasse could be utilized. [Gurieff and Lant (2007]) noted that an effective industrial wastewater treatment process concurrently associated with PHA production would always be a desirable technology. Koller et al. ([2007]) observed that in Europe utilization of regionally arising waste and surplus materials, such as whey for the production of PHA should be forcefully aspired.

Significant improvements in the bioprocessing aspects compared to our previous study (Pramanik et al. [2012]) were achieved in this investigation. The two drawbacks in our earlier report were, first, the lag phases of growth of *Haloarcula marismortui* were prolonged and second, 85% organic carbon remained unutilized in cultures containing 100% pre-treated vinasse. The first limitation was overcome in the present study by using a concentrated inoculum and the second shortcoming was not observed in the present study. Perhaps high growth rate, metabolic versatility and genetic stability of *H. mediterranei* allowed complete utilization of 25% and 50% of pre-treated vinasse. The reduction of BOD_5_ and COD of the pre-treated vinasse were 23% and 35% respectively (Pramanik et al. [2012]), while at least 78% and 80% lowering of BOD_5_ and COD were achieved in this study. Comparing the kinetic parameters, five to six fold improvement in the specific production rate and ten fold rise in the volumetric productivity have been attained in the present study. Thus, *H*. *mediterranei* demonstrated more potential than *H. marismortui* for further development to higher scales.

*H. mediterranei* accumulated 50 wt.-% of poly-3-(hydroxybutyrate-co-8%-hydroxyvalerate) and 73 wt.-% of poly-3-(hydroxybutyrate-co-6%-hydroxyvalerate) from hydrolyzed whey permeate (Koller et al. [2007]; Koller et al. [2008]). In another study, Huang et al. ([2006]) reported 55.6 wt.% accumulation of PHA by the haloarcheon employing extruded rice bran and extruded corn starch. Similarly, Chen et al. ([2006]) applied native corn starch treated by an enzymatic reactive extrusion process leading to 50.8% (w/w) dry cell PHBV content (10.4 mol % HV) in *H. mediterranei.* The present investigation is the first report on the accumulation of 70% of PHBV (12.36 and 14.09 mol % HV depending on the substrate) by *H. mediterranei* utilizing vinasse. Recently the composition of vinasses has been published by Parnaudeau et al. ([2008]). Sugarcane vinasse contains significant amounts of sucrose as well as oxalate, lactate, malate and pyruvate which are ready metabolites to be fed to the tricarboxylic acid cycle. Acetate, valerate, propionate and butyrate may have provided the precursors for HV biosynthesis. The described process may be less expensive than the whey process where costly ultrafiltration step is required. After removing 80% of water (which is also energy intensive) from the sweet skimmed whey, the concentrate is ultra filtrated to obtain the whey permeate (Koller et al. [2007]). Further, an additional pre-treatment step for lactose hydrolysis (enzymatically or chemically) would enhance the cost of production. In the processes employing rice bran and corn starch (Chen et al. [2006]; Huang et al. [2006]), the nutrients of these methods would incur a raw material cost. The outlay would further increase as expensive extrusion machinery and α-amylase will be required. Therefore, among the currently described processes for production of PHA by *H. mediterranei* employing cheap carbon sources, the described method utilizing vinasse is the most economical. Precursors of 3-hydroxyvalerate are known to enhance the 3-HV content in *Haloferax mediterranei*. Starting from the 3-HV content (6.0%) formed by *H. mediterranei* from hexoses of whey sugars as sole carbon source, Koller et al. ([2007]) triggered the content of 3-HV to 22% by addition of 1.0 g/l sodium valerate, a 3-HV precursor. However, the cosubstrates are expensive and add to the production cost as estimated by [Choi and Lee (1999]). Our objective was to utilize a waste product and the effect of many nutrients on 3-HV content was not considered to be studied. Increasing concentration of the vinasse enhances the 3-HV content marginally, probably due to the higher amounts of organic acids available. Huang et al. ([2006]) and Chen et al. ([2006]) did not examine the effect of other additional nutrients on the content of 3-HV.

Salts concentration in the culture medium used for cultivation of *H. mediterranei* should be maintained above 22% (w/v) for optimum cell growth and PHA production (Quillaguamán et al. [2010]). At such a high concentration of salt, the growth of non-halophilic microorganisms is prevented hence allowing a process without strict sterile conditions. The costs of energy required for sterilization can be avoided. Reduction in costs of process piping, instrumentation and insulation as well as saving on electricity for steam generation can be envisaged. A halophilic bacterium, *Halomonas* TD01 isolated from a salt lake in Xinjiang, China was cultivated in an unsterile and continuous fermentation process (Tan et al. [2011]) that opened a new area for reducing the cost in PHA production. The authors produced PHB, the most common PHA while we obtained PHBV that has better mechanical properties than PHB. Tan et al. ([2011]) applied a designed synthetic medium in their unsterile operation while we used waste spent wash direct from the final ethanol distillation column in our unsterile cultivation which is more prone to contamination. We have obtained a product as pure as that marketed by Sigma-Aldrich through a simple isolation process. The isolation methods, purity and molecular characterization of the polymer were not detailed by Tan et al. ([2011]). We carried out an intensive molecular analyses of the polymer obtained through an alternative zero cost carbon source before embarking on a large scale production system which is planned for the future.

The major step of the separation process is the extraction of PHA granules (Jacquel et al. [2008]). Extraction using solvents such as chloroform, methylene chloride, propylene carbonate and dichloroethane resulted in very pure PHA (Ramsay et al. [1994]). This extraction method required large quantities of toxic and volatile solvent, which not only increased the total production cost but also had adverse environmental consequences ([Choi and Lee 1997]). During digestion of non-PHA cellular materials using hypochlorite, severe degradation of PHA was observed. An enzymatic digestion method developed by Zeneca was used for the production of Biopol (PHBV) but the use of expensive chemicals and complex processes did not seem to be economical ([Holmes and Lim 1990]). The extraction step of the described procedure consumed no energy and lesser quantities of chemicals compared to other recovery methods such as solvent and supercritical fluid extraction, digestion by sodium hypochlorite and enzymes as well as mechanical disruption of cell walls by high-pressure homogenization and ultrasonication (Jacquel et al. [2008]). As the PHA granules themselves do not contain much contaminant (Steinbüchel et al. [1995]), highly pure PHA can be produced by processes that break the cell and solubilize cellular material other than PHA. Therefore, a simple digestion method by inexpensive chemicals seems to be the most efficient and economical recovery process. Subsequent purification steps were essential to obtain a high-purity product. Surfactant pre-treatment and hypochlorite digestion under optimized conditions resulted in a very pure PHB with less degradation (Choi and Lee, [1999]) and was applied for post-extraction purification in the present study.

Nonato et al. ([2001]) observed that PHB and related copolymers can be advantageously produced when integrated into a sugarcane mill. This model made extensive use of facilities, materials and surplus energy from the sugarcane industry that would otherwise be wasted or sold at subsistence prices. We believe that amalgamation of PHA production in an ethanol manufacturing unit, as projected in this study is superior to integration in a sugar mill because the PHB production cost in the sugar mill was heavily dependent on sugar prices and accounted for nearly 29% of the final cost. Such concerns should not arise when a highly polluting waste as vinasse is utilized and the process integrated within ethanol manufacture.

The price of production of 1 kg poly-3-(HB-co-HV) was calculated to be € 2.82 at a volumetric productivity of 0.29 g/l h when using hydrolyzed whey as carbon source for *H. mediterranei.* This price is significantly lower than that calculated for the production of PHA by recombinant *E. coli* at ~ € 4.0 (Quillaguamán et al. [2010]). High PHA content, productivity, zero-cost carbon source, low-cost of isolation of a high-purity product and potential for integration into ethanol manufacturing unit would merit further development of this process to higher scales.

## Competing interest

The authors declare that they have no competing interest.

## References

[B1] Bloembergen S Holden D Bluhm TL Hamer GK Marchessault RH Stereochemistry in synthetic β-hydroxybutyrate and β-hydroxyvalerate homopolyesters Macromol 1989 22 1656 1663 10.1021/ma00194a027

[B2] Chen CW Don TM Yen HF Enzymatic extruded starch as a carbon source for the production of poly (3-hydroxybutyrate-co-3-hydroxyvalerate) by Haloferax mediterranei Process Biochem 2006 41 2289 2296 10.1016/j.procbio.2006.05.026

[B3] Choi J Lee SY Process analysis and economic evaluation for poly (3-hydroxybutyrate) production by fermentation Bioprocess Eng 1997 17 335 342 10.1007/s004490050394

[B4] Choi J Lee SY Factors affecting the economics of polyhydroxyalkanoate production by bacterial fermentation Appl Microbiol Biotechnol 1999 51 13 21 10.1007/s002530051357

[B5] Divyashree MS Shamala TR Rastogi NK Isolation of polyhydroxyalkanoate from hydrolyzed cells of Bacillus flexus using aqueous two-phase system containing polyethylene glycol and phosphate Biotechnol Bioprocess Eng 2009 14 482 489 10.1007/s12257-008-0119-z

[B6] Don TM Chen CW Chan TH Preparation and characterization of poly(hydroxyalkanoate) from the fermentation of Haloferax mediterranei J Biomater Sci Polym Ed 2006 17 1425 1438 10.1163/156856206778937208 17260512

[B7] Dong Z Sun X A new method of recovering polyhydroxyalkanoate from Azotobacter chroococcum Chin Sci Bull 2000 45 252 256 10.1007/BF02884685

[B8] Escalona AM Gomis AM Varela FR Procedure for extraction of polyhydroxyalkanoates from halophilic bacteria which contain them US Patent 1996 5 536,419

[B9] Fernandez-Castillo R Rodriguez-Valera F Gonzalez-Ramos J Ruiz-Berraquero F Accumulation of poly(β-hydroxybutyrate) by halobacteria Appl Environ Microbiol 1986 51 214 216 16346972 10.1128/aem.51.1.214-216.1986 PMC238844

[B10] Gurieff N Lant P Comparative life cycle assessment and financial analysis of mixed culture polyhydroxyalkanoate production Bioresour Technol 2007 98 3393 3403 10.1016/j.biortech.2006.10.046 17632000

[B11] Holmes PA Lim GB Separation process US patent 1990 4 910 145

[B12] Huang TY Duan KJ Huang SY Chen CW Production of polyhydroxyalkanoates from inexpensive extruded rice bran and starch by Haloferax mediterranei J Ind Microbiol Biotechnol 2006 33 701 706 10.1007/s10295-006-0098-z 16491353

[B13] Jacquel N Lo CW Wei YH Wu HS Wang SS Isolation and purification of bacterial poly(3-hydroxyalkanoates) Biochem Eng J 2008 39 15 27 10.1016/j.bej.2007.11.029

[B14] Khardenavis AA Suresh Kumar M Mudliar SN Chakrabarti T Biotechnological conversion of agro-industrial wastewaters into biodegradable plastic, poly β-hydroxybutyrate Bioresour Technol 2007 98 3579 3584 10.1016/j.biortech.2006.11.024 17207999

[B15] Khardenavis AA Vaidya AN Kumar MS Chakrabarti T Utilization of molasses spentwash for production of bioplastics by waste activated sludge Waste Manage 2009 29 2558 2565 10.1016/j.wasman.2009.04.008 19500968

[B16] Koller M Atlić A Gonzalez-Garcia Y Kutschera C Braunegg G Polyhydroxyalkanoate (PHA) biosynthesis from whey lactose Macromol Symp 2008 272 87 92 10.1002/masy.200851212

[B17] Koller M Hesse P Bona R Kutschera C Atlić A Braunegg G Potential of various archae- and eubacterial strains as industrial polyhydroxyalkanoate producers from whey Macromol Biosci 2007 7 218 226 10.1002/mabi.200600211 17295410

[B18] Law JH Slepecky RA Assay of poly-β-hydroxybutyric acid J Bacteriol 1961 82 33 36 13759651 10.1128/jb.82.1.33-36.1961 PMC279110

[B19] Liu H Pancholi M Stubbs J III Raghavan D Influence of hydroxyvalerate composition of polyhydroxy butyrate valerate (PHBV) copolymer on bone cell viability and in vitro degradation J Appl Polym Sci 2010 116 3225 3231

[B20] Liu H Han J Liu X Zhou J Xiang H Development of pyrF-based gene knockout systems for genome-wide manipulation of the archaea Haloferax mediterranei and Haloarcula hispanica J Genet Genomics 2011 38 261 269 10.1016/j.jgg.2011.05.003 21703550

[B21] Lu Q Han J Zhou L Zhou J Xiang H Genetic and biochemical characterization of the poly(3-hydroxybutyrate-co-3-hydroxyvalerate) synthase in Haloferax mediterranei J Bacteriol 2008 190 4173 4180 10.1128/JB.00134-08 18408025 PMC2446746

[B22] Martín Santos MA Fernández Bocanegra JL Martín Martín A García García I Ozonation of vinasse in acid and alkaline media J Chem Technol Biotechnol 2003 78 1121 1127 10.1002/jctb.908

[B23] Nonato RV Mantelatto PE Rossell CEV Integrated production of biodegradable plastic, sugar and ethanol Appl Microbiol Biotechnol 2001 57 1 5 10.1007/s002530100732 11693904

[B24] Pant D Adholeya A Biological approaches for treatment of distillery wastewater: A review Bioresour Technol 2007 98 2321 2334 10.1016/j.biortech.2006.09.027 17092705

[B25] Parnaudeau V Condom N Oliver R Cazevieille P Recous S Vinasse organic matter quality and mineralization potential, as influenced by raw material, fermentation and concentration processes Bioresour Technol 2008 99 1553 1562 10.1016/j.biortech.2007.04.012 17582760

[B26] Pramanik A Mitra A Arumugam M Bhattacharyya A Sadhukhan S Ray A Haldar S Mukhopadhyay UK Mukherjee J Utilization of vinasse for the production of polyhydroxybutyrate by Haloarcula marismortui Folia Microbiol 2012 57 71 79 10.1007/s12223-011-0092-3 22258750

[B27] Quillaguamán J Guzmán H Van-Thuoc D Hatti-Kaul R Synthesis and production of polyhydroxyalkanoates by halophiles: current potential and future prospects Appl Microbiol Biotechnol 2010 85 1687 1696 10.1007/s00253-009-2397-6 20024541

[B28] Ramsay JA Berger E Voyer R Chavarie C Ramsay BA Extraction of poly-3-hydroxybutyrate using chlorinated solvents Biotechnol Tech 1994 8 589 594 10.1007/BF00152152

[B29] Steinbüchel A Aerts K Babel W Föllner C Liebergesell M Madkour MH Mayer F Pieper-Fürst U Pries A Valentin HE Wieczorek R Considerations on the structure and bio- chemistry of bacterial polyhydroxyalkanoic acid inclusions Can J Microbiol 1995 41 Suppl 1 94 105 7606669 10.1139/m95-175

[B30] Tamboli DP Kagalkar AN Jadhav MU Jadhav JP Govindwar SP Production of polyhydroxyhexadecanoic acid by using waste biomass of Sphingobacterium sp. ATM generated after degradation of textile dye Direct Red 5B Bioresour Technol 2010 101 2421 2427 10.1016/j.biortech.2009.11.094 20031399

[B31] Tan D Xue YS Aibaidula G Chen GQ Unsterile and continuous production of polyhydroxybutyrate by Halomonas TD01 Bioresour Technol 2011 102 8130 8136 10.1016/j.biortech.2011.05.068 21680179

[B32] Verlinden RAJ Hill DJ Kenward MA Williams CD Piotrowska-Seget Z Radecka IK Production of polyhydroxyalkanoates from waste frying oil by Cupriavidus necator AMB Express 2011 1 11 10.1186/2191-0855-1-11 21906352 PMC3222315

